# Area Coverage Maximization under Connectivity Constraint in Wireless Sensor Networks

**DOI:** 10.3390/s22051712

**Published:** 2022-02-22

**Authors:** Frantz Tossa, Wahabou Abdou, Keivan Ansari, Eugène C. Ezin, Pierre Gouton

**Affiliations:** 1 ImViA Laboratory, University of Bourgogne Franche-Comté, 21000 Dijon, France; pgouton@u-bourgogne.fr; 2 LETIA Laboratory, University of Abomey-Calavi, Abomey-Calavi BP 2549, Benin; eugene.ezin@imsp-uac.org; 3 LIB Laboratory, University of Bourgogne Franche-Comté, 21000 Dijon, France; wahabou.abdou@u-bourgogne.fr; 4 Institute for Color Science and Technology, Tehran 1668836471, Iran; keivan.ansari@gmail.com

**Keywords:** wireless sensor networks, sensors deployment, area coverage, connectivity, genetic algorithm

## Abstract

Wireless sensor networks (WSNs) have several important applications, both in research and domestic use. Generally, their main role is to collect and transmit data from an ROI (region of interest) to a base station for processing and analysis. Therefore, it is vital to ensure maximum coverage of the chosen area and communication between the nodes forming the network. A major problem in network design is the deployment of sensors with the aim to ensure both maximum coverage and connectivity between sensor node. The maximum coverage problem addressed here focuses on calculating the area covered by the deployed sensor nodes. Thus, we seek to cover any type of area (regular or irregular shape) with a predefined number of homogeneous sensors using a genetic algorithm to find the best placement to ensure maximum network coverage under the constraint of connectivity between the sensors. Therefore, this paper tackles the dual problem of maximum coverage and connectivity between sensor nodes. We define the maximum coverage and connectivity problems and then propose a mathematical model and a complex objective function. The results show that the algorithm, called GAFACM (Genetic Algorithm For Area Coverage Maximization), covers all forms of the area for a given number of sensors and finds the best positions to maximize coverage within the area of interest while guaranteeing the connectivity between the sensors.

## 1. Introduction

Wireless sensor networks (WSN) have been the subject of numerous research studies since the first known deployment of the *SOund SUrveillance System (SOSUS)* 1950s [1,2]. Until the 1990s, except for a few radio beacons to route data from a sensor to the central controller expensive and cumbersome cabling was required. New sensor networks appeared in the 1990s, thanks to advances in the field of wireless techniques. As new wireless technologies progress, their range of applications increases. Among these innovative technologies, wireless sensor networks (WSN) have become very flexible and dynamic facets deployed in almost all types of environments whether rural, suburban, or urban [1].

A WSN is a collection of sensor nodes that communicate using wireless technologies. WSNs are made up of small nodes that differ from traditional networks in their communication and detection ranges. Sensor nodes detect the physical phenomena located in the area and transmit the data in collaboration with the receiver node [3]. The data captured by the nodes are routed through a multi-hop routing to a node, considered to be a “collection point”, called sink-node (or sink). The latter can be connected to the network user (via the Internet, a satellite, or another system). A sensor node can be a detection node, a transmission node, a relay node, or a combination of theses nodes.

Initially used in the army, this kind of network is now found in several fields, varying from industrial surveillance to the measurement of environmental data, agriculture, home automation, fire detection, medical sector, etc. [1,4,5,6,7,8,9,10]. These applications are responsible for monitoring a target point or area by recording a reaction and communicating it. Consequently, WSN applications play critical roles whether deployed in industry or in everyday life and must be effective. However, the weak capacities of the sensors both at the level of the detection and communication range constitute one of the first weaknesses of this type of network. The consequences are areas or targets with little or no coverage in the detection region reducing the effectiveness of the network.

Due to the constraining characteristics of the WSNs, one of the first challenges they face is figuring out how to perfectly cover a surveillance area. Coverage and connectivity are the two most fundamental problems of the WSN [6]. One of the solutions that have long been considered to increase the global network coverage has been to increase the number of sensors. However, this solution is not efficient in terms of deployment, coverage, connectivity, and network lifetime. In fact, deployment and collaboration of sensors are some of the major features that directly influence the performance of the WSN. WSN deployment schemes can be classified into two main groups; deterministic and non-deterministic [8,11]. The main performance measures that come into play during the deployment of a WSN include coverage, connectivity, and network lifetime. Among these measures, we focus on coverage and connectivity.

For practical design and use of sensor networks in various application scenarios, coverage depends on several parameters. The coverage reflects how well a sensor monitors a point or an area. In most cases, to obtain maximum coverage a massive number of sensor nodes is required. In [12], coverage is defined as how an area is covered by the sensor or to what extent each point is under of a sensor node. Depending on the kind of application, the coverage can take several forms [11,12,13], including barrier coverage, point coverage and area coverage, which is divided into two types including full area coverage and partial area coverage [3].

In addition to coverage, connectivity is an equally vital element of wireless sensor networks. The network in a defined area is connected if each deployed sensor node has at least one neighboring node with which it is connected. Khoufi, I. et al. [6] explained that two sensor nodes are connected if and only if they can communicate directly (one-hop connectivity) or indirectly (multi-hop connectivity). In [14], network connectivity is defined as the communication capacity between the different nodes of the network where data are transmitted to the base station by a single hop or by several hops. In this transmission process, if a sensor node communicates directly with the base station, it is a single-hop communication. On the other hand, the sensed data are transmitted to the base station via other intermediate sensor nodes, which is called multi-hop communication. When a WSN has single connectivity between sensor nodes, it is called single connectivity or 1-connectivity. The breakdown of a single node in such a scenario can lead to a failure of communication in the network. In the case of k-connectivity (k> 1), despite the failure of one node, the network remains connected. Connectivity is therefore vital for the transmission of sensed data to the base station [6,15]. It has a significant impact on the WSN, as the failure of a link between sensor nodes can result in communication failure in the network.

In real-life situations, usually for cost reasons, the number of sensors is generally restricted but there is a need to cover a large area and provide connectivity. It is also important to cover an area regardless of its shape and the way it is presented geometrically. The issue of coverage and connectivity can be described in various ways. Still, in our case, we are concerned with how to cover more of the area regardless of its geometric shape, with a given number of sensors of the same type while ensuring connectivity. This paper attempts to cover our area of interest (regular and irregular shape) using a genetic algorithm (GA) rather than circular wrapping algorithms [16,17]. Moreover, due to the random deployment of the sensors, causing an overlap, we propose a new fitness function, to know the exact area covered by the sensors.

The rest of the paper is organized as follows: in Section 2, we provide related work on maximum coverage and connectivity. Then, in Section 3, we present the network model by defining the problem and proposing a mathematical model with a detailed objective function. In Section 4, the proposed GA approach is described in detail, and in Section 5, we present and discuss the results. In Section 6, we concludes this paper and gives an overview of some future works.

## 2. Related Works

Optimal positioning of the sensors for accurate measurement and data transmission is vital in wireless sensor networks. For this purpose, node deployment techniques are usually referred to as one of the most important approaches. Although several studies have been carried out to solve this problem, known as the Maximum Coverage Sensor Deployment Problem (MCSDP), there is a continuing requirement to refine the proposed solutions or to propose new ones. To do so, optimization techniques can be used to identify the best node placements that satisfy the desired objectives. In this section, we present some of the recent solutions proposed and based on genetic algorithms.

The coverage issue in WSN was firstly tackled by Yourim Yoon and Yong-Hyuk Kim, who showed that it is NP-hard [18]. They focused on the problem of maximizing the area covered by the sensor with a specified number of sensors. They build their study on *n* static sensors of *k* types, and every type of sensor cover a part of the area with an arbitrary set radius $r_{1},r_{2},...,r_{k}$. They assumed that there is at least one sensor for each sensor type. The goal is to find locations $\left(x_{1},y_{1}\right),\left(x_{2},y_{2}\right),.....,\left(x_{n},y_{n}\right)$ for all *n* sensors that generate a maximum coverage for the area covered. They proposed a new approach to normalizing the problem that could improve the genetic algorithms’ performance. This approach was adapted to MCSDP to combine the GA search with the original solution space. After presenting some GAs (RANDOM, PGA, and MGA) without normalization, they propose OPTGA, which, similar to MGA, uses the Monte Carlo technique with an increased number of random samples to estimate the solutions and reorders the genes of the second parent to minimize the sum of the distances before recombination is applied. To further improve the performance of OPTGA, they merged OPTGA with a local search method called Virtual Force Algorithm (VFA) with some modifications. The VFA defined two concepts: “repulsive force” and “attractive force”. A repulsive force exists between two sensors if their coverage areas overlap; otherwise, an attractive force exists. Based on these two forces, the placement of the sensor is adjusted. In OPTGA, they apply the VFA to each offspring after the mutation step. They call it OPTHGA and show that OPTHGA performs significantly better than OPTGA, even if the OPTHGA algorithms have high computational complexity.

To overcome this pitfall, Ly et al. [19] proposed an algorithm called IGA, which used the individual representation and replaced the Monte Carlo technique [18] with a new technique and introduced a new mutation operator heuristic and dynamic. This has led to a considerable reduction in computational complexity and an increase in the quality of the IGA solution compared to OPTHGA. They introduced a new concept— the overlapping—for designing a new evaluation function, a heuristic technique for initializing population, and a dynamic mutation instead of the static one. The newly-proposed fitness function has shown its tremendous effectiveness in assessing the physical condition of individuals and reducing the complexity of calculations. The heuristic initialization method and the dynamic mutation operator have also improved the quality of their algorithm. They conclude from the experimental results that IGA is better than OPTHGA on all the instances in terms of computation time, quality, and solution stability.

Nguyen Thihanh et al. [3] proposed a solution by reusing the same procedure for the mentioned problem as [18]. They proposed a genetic algorithm called MIGA, based on IGA of [19], to solve the problem of maximizing area coverage in a WSN with heterogeneous nodes. This genetic algorithm is built around the expression of a new chromosome, a custom heuristic initialization, a fusion of the Laplace crossover method (LX) and the arithmetic crossover method (AMXO), and a local search (VFA). They explain this choice of combining the two crossover methods by the fact that in IGA, the crossover operator used makes the offspring generated from the parents strictly follow the genetic information of its parents without the possibility of customizing the parameters. The combining of LX and AMXO guarantees them better results in terms of the quality of the solutions resulting from the crossover. Then, the authors perform the placement by using heterogeneous sensors, and completely excluding overlaps between sensors. By this way, they not only found an optimal positioning for all sensor nodes, but also ensured the maximum of the area coverage as much as possible. To increase the accuracy of the results, they use an exact integral area calculation for the fitness function to compute the area coverage for a set of sensors. The efficiency of their algorithm was then compared to that of IGA, PSO, DPSO, ICS, and CFPA and the results presented show that MIGA offers the best performance in terms of solution quality and stability on a majority of the tested instances.

However, the previous works cited above focus only on coverage and did not respond to the connectivity.

SK. Gupta et al. [20] proposed a genetic algorithm based approach to find potential positions for sensor node placement such that all target points are k-covered and all sensor nodes are m-connected. They presented their linear programming formulation of the problem and then presented their approach based on the genetic algorithm they propose, with an appropriate representation of the chromosomes, the derivation of the fitness function, the selection, crossover, and mutation operators. The authors present a design for chromosome representation and derive a fitness function with three objectives: the use of minimum number of sensor nodes, the coverage, and the connectivity. They represent the chromosome as a string of 0 and 1 and set the length of each chromosome equal to the number of potential positions. Thus for a chromosome, if the value of gene *i*th is 1, it means that a sensor node is placed on the potential position *i*th, and the value of gene 0 indicates that no sensor is placed on the potential position *i*th. Using several WSN scenarios for the experimental results, the authors showed that the proposed algorithm has much better time complexity than other GA-based approaches.

Two evolutionary algorithms are presented and evaluated for the indoor WSN deployment problem in [14]. In the study, authors proposed a multi-objective deployment strategy (MODS) for WSNs based on evolutionary algorithms. The proposed strategy enables the deployment of a wireless sensor network for a smart building application. In the deployment approach, they considered the building constraints (such as walls and doors) that may affect the network communication by using an appropriate propagation model. The proposed WSN deployment method is then formulated as a combinatorial optimization problem by regarding a variety of objectives (energy, connectivity, and network implementation) that compose an exhaustive list of WSN design objectives. That is, unlike other methods, this approach to indoor WSN deployment considers coverage, cost, and connectivity objectives at the same time. The authors have considered constraints such as the multi-wall propagation model that takes into account all the obstacles in the building (i.e., walls, doors, windows, etc.) in order to obtain efficient results. An innovative coding solution, integrating both the cost of the network and the position of the nodes, was proposed. Indeed, in this study, the authors have coded the chromosome in such a way that it can integrate several pieces of information in a single bit (node coordinates and network cost). Finally, a comparative study between two evolutionary algorithms (classical GA and NSGA-II) was also presented to identify the use case of each.

Subash Harizan and Pratyay Kuila in [21], proposed an improved genetic algorithm-based approach for energy-efficient scheduling of sensor nodes. A collection of N sensor nodes, $S=S_{1},S_{2},S_{3},.......,S_{N}$, is randomly spread within a region of interest to continuously or periodically monitor a set of *K* targets, $\psi =\psi_{1},\psi_{2},\psi_{3},.......,\psi_{k}$. Therefore, the proposed algorithm will select a set of minimum number of active sensor nodes from *S* to provide complete coverage of the target points and also maintain connectivity between the activated sensor nodes and the base station. During scheduling, sensor nodes of higher energy levels are preferred so that they can provide the service for maximum number of rounds. Thus, this is on of the aspect on which they are evaluated by the fitness function. Indeed, the authors derived an efficient fitness function to deal with four conflicting objectives with are to activate a minimum number of sensor nodes among the densely deployed sensor nodes, provide full coverage at all target points, maintain connectivity between the activated active sensor nodes and the base station, and ensure the selection of the active sensor nodes with a higher level of residual energy. Additionally, unlike the traditional GA, the authors have introduced another way of conducting the mutation operation to gain performance improvement and speed up the convergence of the proposed algorithm. In this mutation operation, instead of randomly returning the gene value, redundant or unused activated sensor nodes are found and turned off without affecting the network coverage and connectivity. Finally, the authors present several simulation cases of the approach and compare its performance to NSGA-II, traditional GA, and the GreedyCSC algorithm in terms of the number of sensor nodes selected in a schedule, the energy consumption of the network, and the number of turns required for the first sensor node to be completely without energy.

ZainEldin Hanaa et al. in [22] proposed a GA-based deployment technique to maximize the coverage of randomly spread nodes in an area of interest, which is an M×N grid with a collection of homogeneous wireless sensor nodes $S=S_{1},S_{2},S_{3},...,S_{n}$. They introduced an improved dynamic deployment technique based on genetic algorithm, called IDDT-GA, to maximize the area coverage by reducing the number of sensor nodes in the random deployment. Indeed, by reducing the number of sensor nodes in this way, the authors decrease the area of overlapping between sensor nodes and consequently increase the area covered by the sensors. The approach introduced in their paper uses the variable length coding notation by a two-point crossover and allows them to guarantee 1-connectivity between sensor nodes. In fact, the two-point crossover allows the chromosome length to be adaptive and the algorithm to search for the minimum number of sensor nodes that can cover the area. Thus, IDDT-GA uses a sufficient number of nodes to cover the entire region. The authors will then use random deployment to produce regions of varying intensity and regions with high density, while others, of lower intensity but interesting data, may be lost due to lack of optimal coverage. To compensate this, they add a mobility function to eliminate coverage gaps and increase the coverage of the area. The authors present several simulation results and comparisons with other algorithms which attest to the effectiveness of their proposal.

Njoya, A.N. et al. [23] addressed the target coverage as in [20]. They proposed a hybrid approach that ensured sensor deployment on a grid for coverage targets while considering connectivity. The authors proposed a sequential hybrid approach based on three algorithms. Based on the location of targets, the location of the sink, and the number of targets, the first placed the sensors to all targets were covered. The second removed redundancies from the placement algorithm to reduce the number of sensors deployed. Finally, to establish the connectivity between sensors, the third one, based on the Traveling Salesman Problem (TSP) with the genetic algorithm, generates a tree that gives a minimal path that links deployed sensors and sink. An essential aspect of their placement algorithm is the deterministic initialization at the start of the deployment algorithm that differs from those that do it randomly.

Later, authors in [24] tackle fault tolerance and connectivity problems in WSNs. They focused their work on the restoration of connectivity and proposed a simple and efficient algorithm to accomplish movement-based *k*-connectivity restoration that divides the nodes into the critical, which are the nodes whose failure reduces *k*, and non-critical groups. The algorithm picks up and moves the non-critical nodes when a critical node stops working. This algorithm (PINC) moves a non-critical node with minimum movement cost to the position of the failed mote. The use of ultrasonic waves, or the use of a unique strength indicator received, allows them to identify obstacles, so that nodes can detect and ignore links passing through obstacles. This simplifies the communication and movement model. This way, nodes with communication links can move directly to each other’s position. The authors have proven the accuracy and complexity of their algorithm by stating four theorems that they prove. They also show, from their complexity analysis, that the time complexity of the proposed PINC algorithm is better than its counterparts. They then implemented the PINC algorithm on a small scale on a testbed of Kobuki robots and IRIS sensor motes.

More recently, the authors in [25] have discussed connectivity estimation approaches for internet of things enabled wireless sensor networks. In this study, the authors review connectivity estimation approaches, describe their main ideas, and explain how they work by illustrating example networks. As, in WSN, connectivity is generally obtained in two cases (*k* = 1 and *k* > 1), the authors divided the connectivity estimation problem into two categories. For each case, they presented the algorithms for detecting cut vertices and bridges and explain in detail how these algorithms work. For the case *k* = 1, several algorithms such as BFS, I-PRITCHARD, ENDBRIDGE, MILIC and E-MILIC, ABIDE, and DENCUT have been presented with each their specificities. For the case *k* > 1, algorithms such as SECO, NIKE, BFSK, PACK, DECK, and KEIP have been discussed.

To the best of our knowledge, with the exception of [19], for overlaps, the studies cited above and encountered in the literature perform the deployment based on non-overlapping cases and use M×N regular shape zones. In this paper, we introduce a deployment study for the maximum coverage considering overlaps while providing connectivity. Consequently, we have focused on the coverage of the most possible area with a set of sensor nodes, regardless of the geometric shape of the area concerned. The maximum coverage problem addressed in this paper is related to the calculation of the area covered by the deployed sensor nodes. Our work is based on GA and concentrates on:

- the choice of optimal sensor positions;
- finding the actual area covered by the sensors taking into account the overlap;
- covering all types of area (regular shape or not);
- deriving an efficient objective function with two main objectives: maximizing coverage in the observed area and minimizing coverage outside of the observed area with constraints to ensure connectivity.

## 3. System Model

### 3.1. Network Model and Assumptions

This article examines the double problem of maximizing area coverage while ensuring connectivity in the WSN as formulated above. We consider a WSN application scenario where a set of N sensor nodes, $S=\{s_{1},s_{2},s_{3},.....,s_{N}\}$, are randomly deployed within a region of interest. We are dealing with many types of regions, so the area to be covered with sensors may or may not be regular in shape. We assume that all the sensors are homogeneous and that the computing and power capacities of all these devices are the same. We also assume that each sensor has an omnidirectional antenna and that the detection range $r_{s}$ and the communication range $r_{c}$ are equal and arbitrary. The detection area of each sensor is modeled by a circle; the center of the circle indicates the position of the sensor and its radius indicates its detection and communication range. The sensing range parameter is the same as in the literature, [18,20,26] and the cover model is boolean. The boolean disk coverage model is the most widely used in the literature [3,18,20,26,27,28] and is defined by Equation (1):

(1) $$f\left(s,p\right)=\left\{\begin{array}{cc} 1 & \;\mathrm{if}\;d\left(s,p\right)\leq r_{s}\\ 0 & \;\mathrm{otherwise}. \end{array}\right.$$

where $r_{s}$ is the sensing range and d(s,p) the Euclidean distance from the sensor *s* to a point *p* is given by Equation (2):

(2) $$d\left(s,p\right)=\sqrt{{\left(s_{x}-p_{x}\right)}^{2}+{\left(s_{y}-p_{y}\right)}^{2}}$$

### 3.2. Coverage and Connectivity Model

**Figure 1:**
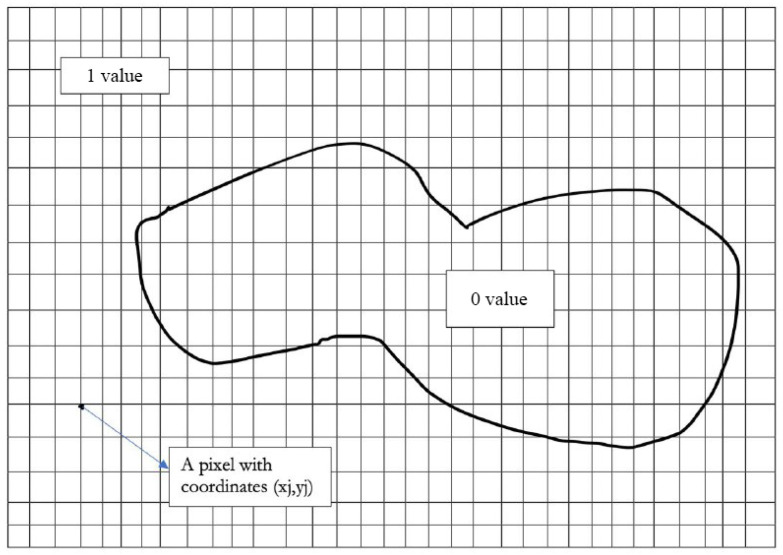
Area to cover in a grid.

Figure 1 shows a map that represents an area of interest. This area is represented in a grid containing pixels. The pixel is the basic unit of programmable color on a computer display or in a computer image. The “1 value” area represents the area where the pixels are at 1 while “0 value” area represents the area where the pixels are at 0. There are n×n pixels in the grid. Suppose that the map consists of the number *m* of pixels. The pixels inside the map are set to 0 while everywhere else is set to 1, as shown in Figure 1.

**Figure 2:**
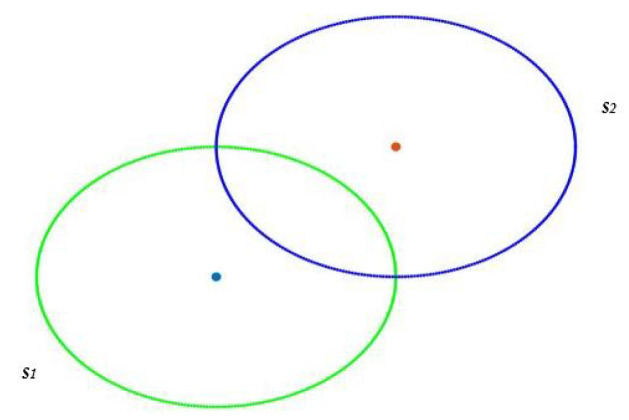
Two unconnected sensors.

**Figure 3:**
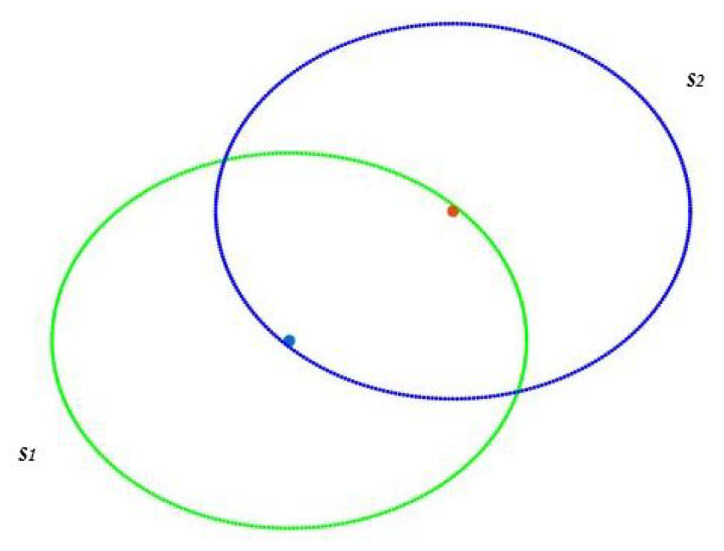
Two connected sensors.

The *i*th sensor has coordinates $\left(x_{i},y_{i}\right)$ such that most pixels of the map are located inside the circle with center $\left(x_{i},y_{i}\right)$ and radius $r_{s}$. This means that pixels of the map would be covered by at least one sensor. In addition, we expect pixels outside the region of interest to be uncovered, or as little covered as possible. Finally, full connectivity must be guaranteed. This means that whatever pair of sensors is chosen, there is at least one path that connects them. Sensor nodes communicate within communication range $\left(r_{c}\right)$ to exchange data until reaching the sink. The network said to be connected if there is at least one path (*k* = 1) between each sensor node and the sink. Moreover, if there are multiple and different paths between each sensor and the sink, this is called k-connectivity where k>1 [15]. Two sensors $s_{1}$ and $s_{2}$ are said to be unconnected if the Euclidean distance between $s_{1}$ and $s_{2}$ is greater than $r_{c}$ (Figure 2) and connected if this distance is less or equal to $r_{c}$ (Figure 3).

### 3.3. Formulation of Linear Programming (LP)

We formulate the above problem as the following mathematical optimization problem:

1. The map to be covered is represented in Figure 1 by the “0 values” zone, and we call it *A*.
2. *M* denotes the set of pixels which are in the map.
  (3) $$M=\left\{\begin{array}{c} j/j\in A⟺j=0 \end{array}\right\}$$
3. $M^{\prime }$ denotes the set of pixels which are outside of the map.
  (4) $$M^{\prime }=\left\{\begin{array}{c} j/j\notin A⟺j=1 \end{array}\right\}$$

The coverage and connectivity problem can be stated as follows. Given *A*, we need to place the sensor nodes to cover most of *A* while ensuring that the network is fully connected.

Let $Z_{ij}$, $Z_{ij}^{\prime }$ and $C_{ij}$ be the Boolean variables defined as follows:

$Z_{ij}$ denotes the set of pixels in the map which are covered by a sensor $s_{i}$

(5) $$Z_{ij}=\left\{\begin{array}{c} 1\;\;if\;j\mathrm{th}\;pixel\;of\;map\;is\;covered\;by\;i\mathrm{th}\;sensor,\;\forall \;j\in M\\ 0\;\;otherwise. \end{array}\right.$$

$Z_{ij}^{\prime }$ denotes the set of pixels outside of the map which are covered by a sensor $s_{i}$

(6) $$Z_{ij}^{\prime }=\left\{\begin{array}{c} 1\;\;if\;j\mathrm{th}\;pixel\;outside\;of\;map\;is\;covered\;by\;i\mathrm{th}\;sensor,\;\forall \;j\in M^{\prime }\\ 0\;\;otherwise. \end{array}\right.$$

$C_{ij}$ denotes the connectivity between sensor $s_{i}$ and $s_{j}$

(7) $$C_{ij}=\left\{\begin{array}{c} 1\;\;if\;d\left(s_{i},s_{j}\right)\leq r_{s}\\ 0\;\;otherwise. \end{array}\right.$$

where $d(s_{i},s_{j})$ is the Euclidean distance and its give by Equation (2).

Then, the formulated linear programming problem is given by Equation (8):

(8) $$Object=\left\{\begin{array}{c} Maximize\;Z_{1}=\sum_{i=1}^{N}\sum_{j\in M}Z_{ij}\\ Minimize\;Z_{2}=\sum_{i=1}^{N}\sum_{j\in M^{\prime }}Z_{ij}^{\prime } \end{array}\right.$$

## 4. Genetic Algorithm

Various metaheuristic algorithms exist in the literature with many general foundations. They implement a form of stochastic optimization so that the solution found depends on the set of random variables generated by the search over a large set of feasible solutions. Metaheuristics can often find reasonable solutions with less computational effort than iterative methods or simple heuristics. Therefore, they are practical approaches for optimization problems. In the field of combinatorial optimization, Padula and Kincaid [29] described several combinatorial optimization methods such as Tabu Search (TS), Simulated Annealing (SA), and Genetic Algorithm (GA) for actuator/sensor placement. Among these methods, we have conducted our study using the genetic algorithm.

In 1859, the naturalist Charles Darwin published his famous book *The Origin of Species*, which presented a theory to explain the phenomenon of evolution. About 100 years later, John Holland and his colleagues and students at the University of Michigan were inspired by it and tried to implement evolutionary systems based on natural selection artificially. This work then led to Genetic Algorithms, belonging to the family of evolutionary algorithms, which are used to obtain approximate solutions for certain complex optimization problems when there is no exact method (or the solution is unknown) to solve them in a reasonable time [20,21,22,23]. The novelty introduced by this group of researchers was the consideration of the crossover operator and the mutation [30].

The genetic algorithm begins with a randomly generated population of possible solutions [20]. A solution is represented by a set of genes called a chromosome or individual. A fitness function evaluates each individual to determine its quality. At the end of the generation of the initial population, three operations are performed. These are selection, crossover, and mutation [20,21]. In the selection phase, a set of probable solutions is acquired from the initial population. Then, two chromosomes (parents), chosen at random, are used to create two other chromosomes ( offspring) by the operation of crossover in which the parent exchange their genetic information. The offspring then undergo the process of mutation in order to produce a better solution. However, the mutation process does not always produce a better solution. It may even alter the mutated solutions. Once the mutation is complete, the offspring are evaluated by the fitness function, and their values are compared to those of all the chromosomes of the previous generation. If the current offspring have higher fitness values, they replace the parents in the new population. The crossover and mutation operation is iterated until the final criteria, which can be a fixed number of generations or a chromosome of the desired quality, is achieved.

### 4.1. Chromosome and Initial Population

**Figure 4:**
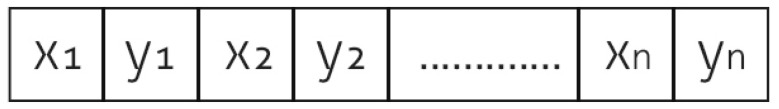
Chromosome structure.

A chromosome represents a complete solution to the problem to solve. In our case, we represent the chromosome as a string of coordinates $\left(x_{i},x_{j}\right)$. An individual is encoded as an array of sensor node coordinates; each array member represents the corresponding sensor’s location in the surveillance region. The length of each chromosome is twice the number of deployed sensors. Thus, if we deploy 20 sensors, each chromosome will be constituted with a string of 20 possible positions (coordinates), that is, 40 elements. Consider a WSN with 20 sensors $S=s_{1},s_{2},s_{3},.,s_{20}$. The length of a chromosome is thus 2×20, which corresponds to the number of potential positions as shown in Figure 4.

As said above, the initial population is a randomly generated set of chromosomes in which each chromosome is a collection of coordinates. We use a function to return a row vector containing *N* unique integers selected randomly from 1 to *k*. The initial population generation algorithm is shown in Algorithm 1.
**Algorithm 1:** Generation of the initial population **Require:**  Number of sensors, N  Size of the initial population, pop size  Grid_Number, k **Ensure:** Initial Population, initPop   1: initPop = [ ];   2: **for** i = 1 to popsize **do**   3:      initPop = Grid(randperm(k,N),:);   4: **end for**

The Grid function first allows us to have a grid background as shown in the representation made in Figure 1. The grid obtained will depend on the loaded map. The center of any sensor can only be placed at the intersection of two grid lines, thus giving the *x* and *y* coordinates of each sensor. There are therefore as many possibilities of choice of positions as of line intersections on the grid. This intersection number is provided in the variable Grid_number=k. Therefore, in line 3, the program seeks to position the *N* sensors randomly on the *k* possible intersections, by traversing the grid from the first random position chosen until the end of the grid.

### 4.2. The Operators

#### 4.2.1. Selection

The selection phase defines the ability of an individual to participate in reproduction in a given generation. In most cases, individuals with a higher fitness value are chosen more than the rest. Different selection processes are available in the literature, such as Roulette wheel, Rank selection, Tournament selection, or Boltzmann selection [20,31]. In our case, the selection of individuals to be subjected to crossover and mutation is performed by rank. Ranks are assigned to individuals according to their fitness value so that each individual has a chance to be selected according to its rank. The rank-based selection method reduces the risk of premature convergence of the solution to a local minimum [31].

#### 4.2.2. Crossover

**Figure 5:**
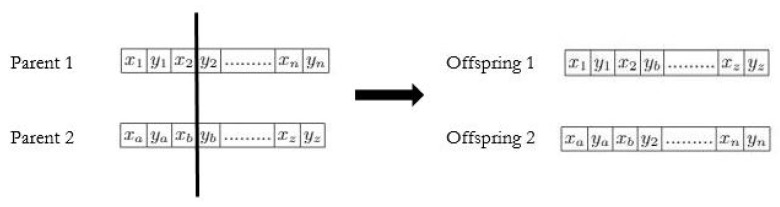
Single point crossover.

After the selection operation, the crossover is applied on the randomly chosen two parent chromosomes from the current population to produce new child chromosomes. There are various types of crossover such as single-point, two-point, k-point, uniform, partially matched, order, precedence preserving crossover, shuffle, reduced surrogate, and cycle [21,31]. In our case, we use a single-point crossover. A random crossing point is selected, and the genetic information of two parents beyond that point will be swapped with each other. Figure 5 shows the genetic information after swapping.

#### 4.2.3. Mutation

The mutation is an operator that maintains the genetic diversity from one population to the next population. The well-known mutation operators are displacement, simple inversion, and scramble mutation [31].

#### 4.2.4. Evaluation Function

The fitness value of a chromosome represents its level of quality based on the objectives. In the proposed work, the objective is to place the sensors within the area of interest to guarantee the maximum coverage inside the area of interest, the minimum outside the area, and maintain full connectivity between the sensors. The fitness function, to be maximized, was defined as the exact percentage of area covered within the area of interest.

We build the fitness function depending on the following parameters described as follows.

1. Guarantee the maximum coverage inside the area of interest. Therefore, first objective will be as follows:
  Objective 1:
  (9) $$Maximize\;Z_{1}=\sum_{i=1}^{N}\sum_{j\in M}Z_{ij}⟺Minimize\;Z_{1}^{\prime }=\sum_{i=1}^{N}\sum_{j\in M}\left(1-Z_{ij}\right)$$
2. Have the least coverage outside.
  Objective 2:
  (10) $$Minimize\;Z_{2}=\sum_{i=1}^{N}\sum_{j\in M^{\prime }}Z_{ij}^{\prime }$$
3. Under the connectivity constraint as shown in Equations (7) and (8).

To implement Equations (9) and (10), we estimate the area covered by treating each sensor node as an image and provide a way first to delineate the area of interest and then calculate the exact coverage. We simulate a two-dimensional area as a background with a number of 0. This area is the smallest possible area encompassing our area of interest where the sensors should be deployed. The 0 number is determined by the resolution initially chosen. For instance, an area of 10 × 20 with a resolution of 0.5 will provide a 20 × 40 array of 0 s in the matrix. The size of the circle materialized by the sensor is related to the radius defined in the chromosome. Each chromosome gives the *x* and *y* axes of the location of the circles in the defined rectangular area. The coordinates *x* and *y* are for the first generation created randomly. They then vary with each generation to obtain the best possible locations. Each time a sensor is placed, the circle formed by this sensor is automatically filled with 1. If the sensors overlap, the 1 is written and counted only once. This ensures that accurate coverage is found. If sensors or parts of sensors are deployed outside the area of interest, the 1 s that are outside are not counted. Finally, the program counts the number of 1 s inside the area of interest. The coverage rate is the ratio of 1 s to the initial number of 0 s. The Algorithm 2 summarizes how the final result is reached.
**Algorithm 2:** Coverage function1:Simulation of a two-dimensional background *S* with a number of 0;2:Counts the number of 0 inside *S*;3:s←numberof0;4:Circular stamp is the square of 0 which is entirely filled by the circle formed by 1;5:The coordinates *x* and *y* of the position of the circles inside *A* are given by each chromosome;6:Put the center of a stamp on the position of defined circles in the chromosome;7:Count the number of 1 inside *A*;8:**if** Overlap between circle **then**9:    Count once 1 in the overlapped area;10:**else**11:    Count number of 1;12:**end if**13:**if** Circle goes outside of *A* **then**14:    Count only the one that are inside;15:**else**16:    Count number of 1;17:    b←numberof1;18:**end if**19:Coverage=b/s×100;

To establish and respect the connectivity constraint, as present in Equation (7), we proceed as follows: with respect to each solution, there are pairs of $[x_{i},y_{i}]\;for\;i=1,...,N$ and a unique radius *r*. $\left[x_{i},y_{i}\right]$ is the location of the *i*th sensor deployed. We consider the points $x_{i}$ as the set of vertices of a graph named *G*. Then we generate a square matrix of size N×N called *C*. The matrix *C* is considered as the adjacency matrix of the graph *G*. $c_{ij}$ is equal to 1 if the distance between the position $\left[x_{i},y_{i}\right]$ and the position $\left[x_{j},y_{j}\right]$ is less than or equal to the value *r*, and 0, otherwise. We now look for the connected components of the graph *G*. A connected component is a group of sensors that are connected to each other. Each connected component is put into a variable as a batch, and each batch is assigned a number. This number indicates to which component each node of the graph belongs. If the batches have only one number for all nodes, it means that *G* has only one connected component and that all sensors are connected to each other so that *G* is fully connected.

**Figure 6:**
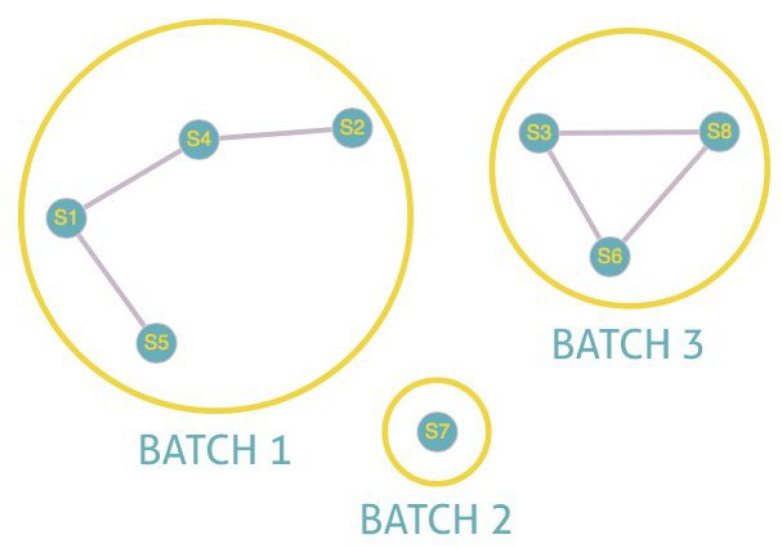
Unfully connected.

**Figure 7:**
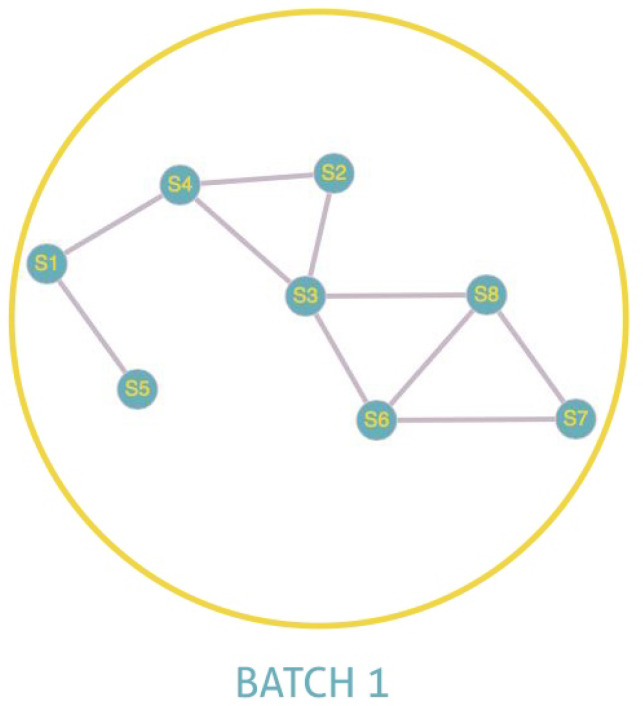
Fully connected.

Figure 6 and Figure 7 illustrate the explanation given above. In these illustrations, we will consider that two sensors are connected if there is a link between them and not connected otherwise. In Figure 6, we note that $S_{5}$, $S_{1}$, $S_{4}$, and $S_{2}$ are connected between them. The same is true for $S_{6}$, $S_{3}$, and $S_{8}$. $S_{7}$, being alone, is by default connected to itself. The program, therefore, creates three batches with a number for each batch. All the nodes belonging to batch one will have the number 1 and so on. The existence of more than one batch, and therefore of several numbers, means that the graph is not fully connected. Indeed we have three connected components for Figure 6. On the other hand, in the case of total connectivity, as illustrated in Figure 7, the program will put all the sensors in the same batch. As there is only one batch number and therefore only one connected component, we can deduce that the graph is fully connected.

## 5. Experimental Results and Discussions

### 5.1. Experimental Results

We performed experiments on the proposed algorithm using MATLAB x64 on a system with Core (TM) i7, 3.6 GHz, 8 GRAM, Windows 10 as a platform. A code was developed to implement the algorithm, and selected simulation cases have been executed to demonstrate the efficiency of the proposal. The algorithm automatically runs several times, changing the radius of the sensors or not and calculating the corresponding coverage inside the area of interest with the connectivity constraint. In the end, the algorithm chooses the best result. We have performed several simulations in several cases that show the algorithm’s effectiveness in both deploying and maintaining connectivity. All cases are based on actual maps of cities or parts of cities taken from Google Maps. For the cases illustrated and detailed below, we use the parameters shown in Table 1.

**Figure 8:**
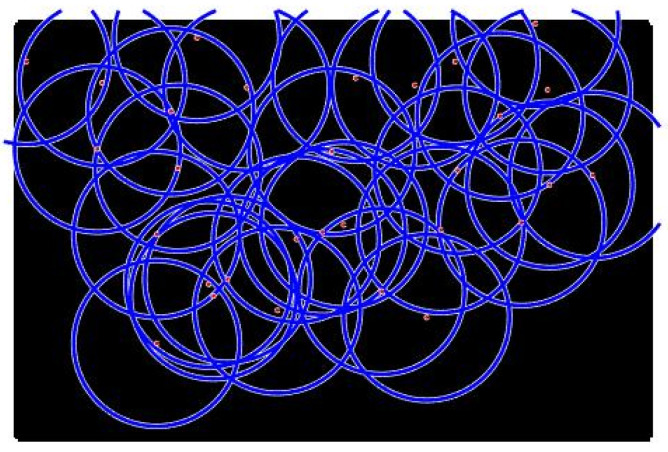
Final deployment.

**Figure 9:**
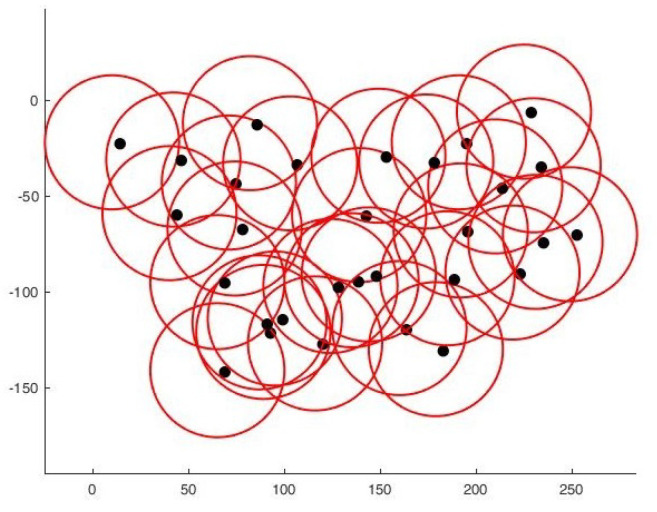
Sensors connectivity scheme.

**Figure 10:**
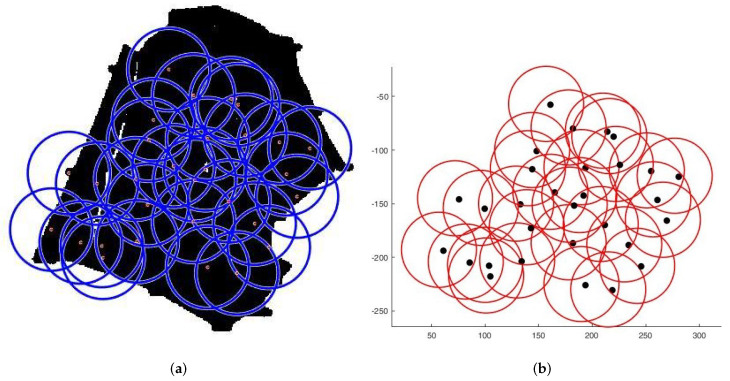
Deployment on first area. (**a**) Final positioning. (**b**) Connectivity scheme.

**Figure 11:**
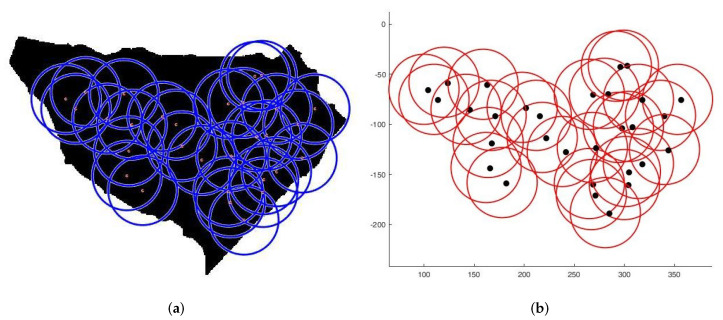
Deployment on second area. (**a**) Final positioning. (**b**) Connectivity scheme.

**Figure 12:**
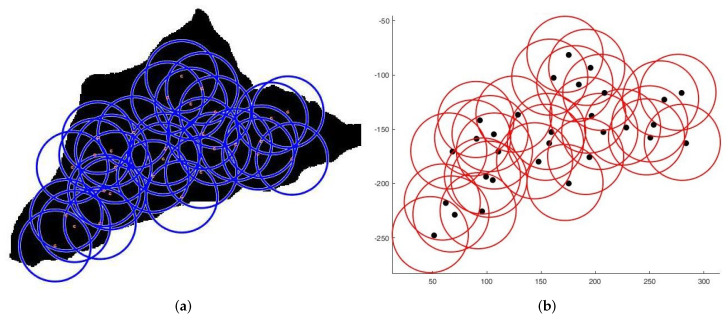
Deployment on third area. (**a**) Final positioning. (**b**) Connectivity scheme.

**Figure 13:**
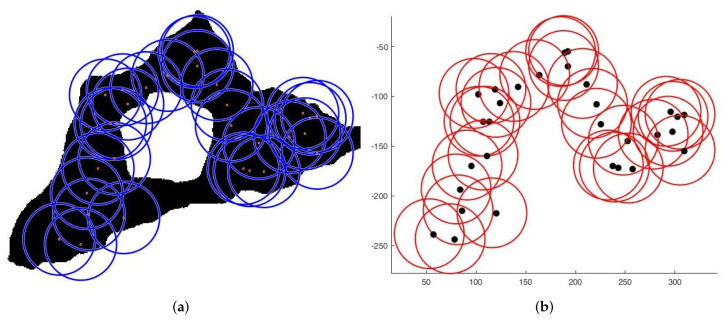
Deployment with one “no interest” area. (**a**) Final positioning. (**b**) Connectivity scheme.

**Figure 14:**
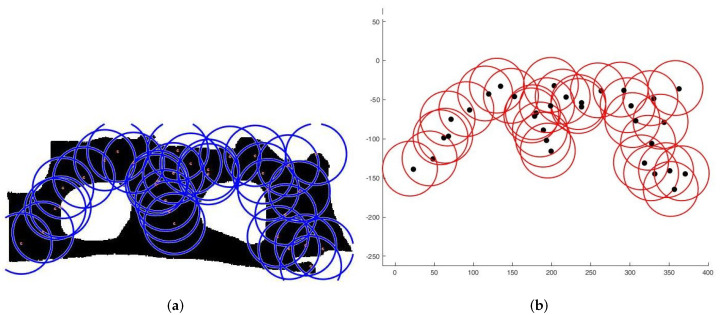
Deployment with two “no interest” area. (**a**) Final positioning. (**b**) Connectivity scheme.

- **Case 1**:
  For the first tests, we deploy sensors on a regularly shaped area of dimensions L×l. The objectives remain unchanged. At the beginning of the test, we have an initial and random deployment of the sensors. At every iteration, the location of the sensors is displayed, and at the end, the best population and the final position of sensors (Figure 8) are given, based on the fitness value and the connectivity constraint. Figure 9 shows the sensor connectivity scheme.
- **Case 2**:
  For the second test, we deploy sensors on three irregularly shaped areas, keeping the same objectives. Figure 10, Figure 11 and Figure 12 show the final position of the sensors and the sensor connectivity scheme corresponding in each case.
- **Case 3**:
  For the third set of tests, we create an area of "no interest" on the map using the last deployment area. The goal here is to deploy sensors while maintaining the previously stated objectives. To illustrate, in the case of deployment in a city, it would be useless to deploy sensors in a lake or an area empty of houses. Figure 13 and Figure 14 show the final position of the sensors in each case and the sensor connectivity scheme corresponding to each final positioning.

### 5.2. Discussion

In order to evaluate the performance of the proposal, an extensive simulation is performed considering many different network scenarios as described in the previous section. All scenarios considered represent actual maps and are simulated with a grid-based detection field of at least 300×300. In all network scenarios, sensor nodes are randomly placed in the area during the first deployment. In our simulation, we took an initial population of 150 chromosomes. However, there are no universal rules for defining the population size. The end criterion is taken as the maximum number of iterations which in our case is 400. However, we observe that the algorithm converges before reaching the maximum number of iterations. The detection and communication range of the sensor nodes is identical, as specified above, and is equal to 35 m. The simulations are performed 30 times for each scenario, and the average result is considered.

First, we show the relationship between the coverage rate and the number of sensors in Table 2 below. As connectivity is always acquired, it remains constant with radius. Therefore, in this table, two variables come into play for the same radius, namely the coverage rate and the rate coverage out of the area of interest.

By varying the number of sensors, we observe the percentage of coverage inside the area of interest and the percentage of coverage outside. As connectivity is always acquired, it remains constant with radius. By combining these values, we notice that the variation of the percentage of coverage when we go from 40 sensors to 60 or from 60 to 80 is not significant (1.7%). The right compromise in terms of nodes and coverage is between [40–60–80].

Then, we ran several test instances varying the population size and the number of iterations and then considered the average of the results. By varying the population size in the tests, we can see that we achieve almost 99% coverage with a population size of 50. We then varied the number of iterations and noted that we achieved convergence with a coverage rate above 98% for 200 iterations.

We also provide a time performance of the algorithm using the parameters in Table 1. We vary the number of generations and we observe the time taken by the algorithm to propose a first good solution, which for us comes down to a first connected deployment. We note this duration DFGS (duration for first good solution). Then, we observe the total time taken to go through all the generations. The unit of time is the second and the results are presented in Table 3.

We observe that at 20 generations, it takes only 8 s for the algorithm to propose a first good solution to the third generation and 48 s to go through the 20 generations by improving the proposals. At 40 generations, it takes 31 s for the first good solution to the 13th generation and 105 s in all. At 80 generations, it takes 100 s for the first good solution to the 37th generation and 213 s in all. At 160 generations, it takes 105 s for a first good solution at the 41st generation and 420 in all. At 320 generations, it takes 89 s for a first good solution to the 34th generation and 840 s in all. Finally, at 640 generations, it takes 34 s for the first good solution to the 14th generation and 1800 s in all.

**Figure 15:**
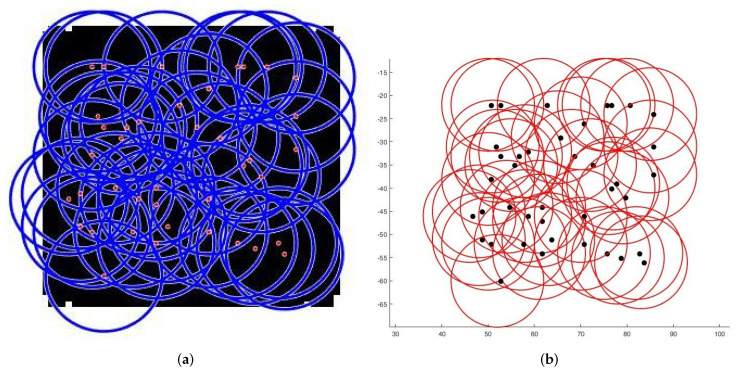
Deployment on a 100×100 square. (**a**) Final positioning. (**b**) Connectivity scheme.

Finally, we compare our proposal with [22] one of the recent papers working on area coverage and connectivity. As these authors deal with the area coverage problem, excluding or minimizing overlaps, a strict comparison is difficult. Secondly, to the best of our knowledge, none of the authors encountered in the literature treat the problem from any other angle than regular forms. However, by comparing with [22], and based on the results provided by the authors; we can see that our proposal provides a better solution in terms of coverage/area covered ratio. Indeed, for an area of 100×100, they reach 97.25% of coverage with a number of sensors between 30 and 50, while for a larger area, we go beyond this percentage with as many sensors. Table 4 summarizes a brief comparison made between the two works and Figure 15 shows the results we obtained in terms of deployment and connectivity scheme by using their parameters.

## 6. Conclusions

This paper proposed a GA-based scheme to find the best positions for sensor node placement in wireless sensor networks, respecting the coverage and connectivity between the sensor nodes. The need for such a method has been demonstrated when an entire area is of interest in a study. We first presented the linear programming formulation of the problem. We then explained the representation of our chromosomes, selection, crossover, mutation, and finally, the derivation of the fitness function through the algorithm based on the calculation of the percentage of coverage and the one based on the connectivity between sensors. Our proposal has been extensively simulated by varying the number of test cases and using several scenarios. Unlike the other methods presented in the literature review, ours is placed in an actual deployment situation and considers the overlap between one or more sensors to compute and give the exact maximum coverage rate, focusing on the areas of interest while maintaining full connectivity. Several tests have been performed on areas of different shapes, ranging from regular to irregular. These tests show that we can achieve a coverage rate of up to 95%. The results show that the algorithm is efficient and that the proposed approach satisfies the desired objectives.

The proposal has a wide range of applications in smart cities, for example. However, in this paper we have not addressed the issue of the connectivity disruption or energy efficiency in our deployment. In our future work, we will develop an even more efficient scheme that will consider the connectivity disruption, energy, redundancy, and finally, the routing of the detected data.

## Figures and Tables

**Table 1:** Simulation parameters.

Parameter	Value
The number of executions on one instance	30
Population size	150
Number of generation	400
Crossover rate	0.7
Mutation rate	0.01
Number of sensors	30
Radius	35

**Table 2:** Coverage percentage.

Number of Sensors	% Coverage in	% Coverage out
10	43.84	0
20	82	0
40	96.52	2.1
60	98.22	1.78
80	99.9	0.1

**Table 3:** Simulation time.

Number of Generation	DFGS	Total Duration
20	8	48
40	31	105
80	100	213
160	105	420
320	89	840
640	34	1800

**Table 4:** Comparison parameters.

Parameters	GAFACM	IDDT-GA
Deployment on regular form	Yes	Yes
Deployment on irregular form	Yes	No
Deployment with overlapping	Yes	Minimizing overlaping
Size of deployement area	100×100	100×100
Number of sensors	40	45
Sensing range	10	10
Population size	30	30
Number of generation	500	500
Percentage of crossover	0.7	0.7
Percentage of mutation	0.01	0.01
Coverage rate	100%	97.25
Full connectivity	Yes	Depends on the number of sensors

## Data Availability

Not applicable.
